# Comparison of coronary computed tomography angiography image quality with high- and low-concentration contrast agents (CONCENTRATE): study protocol for a randomized controlled trial

**DOI:** 10.1186/s13063-016-1441-y

**Published:** 2016-07-15

**Authors:** Dong Jin Im, Yun-Hyeon Kim, Ki Seok Choo, Joon-Won Kang, Jung Im Jung, Yoodong Won, Hyo Rim Kim, Myung Hee Chung, Kyunghwa Han, Byoung Wook Choi

**Affiliations:** Department of Radiology and Research Institute of Radiological Science, Severance Hospital, Yonsei University College of Medicine, Seoul, Republic of Korea; Department of Radiology, Chonnam National University Hospital, Chonnam University Medical School, Gwangju, Republic of Korea; Department of Radiology, Pusan National University Yangsan Hospital, Pusan National University School of Medicine, Pusan, Republic of Korea; Department of Radiology and Research Institute of Radiology, Asan Medical Center, University of Ulsan College of Medicine, Seoul, Republic of Korea; Department of Radiology, Seoul St. Mary’s Hospital, The Catholic University of Korea, Seoul, Republic of Korea; Department of Radiology, Uijeongbu St. Mary’s Hospital, Catholic University of Korea, Uijeongbu, Republic of Korea; Department of Radiology, Yeouido St. Mary’s Hospital, Catholic University of Korea, Seoul, Republic of Korea; Department of Radiology, Bucheon St. Mary’s Hospital, Catholic University of Korea, Bucheon, Republic of Korea; Department of Radiology, Severance Hospital, Yonsei University College of Medicine, 50-1 Yonsei-ro, Seodaemun-gu, Seoul 03722 Republic of Korea

**Keywords:** Computed tomography, Coronary computed tomography angiography, CCTA, Coronary artery disease, CAD, Contrast agent, Radiation

## Abstract

**Background:**

With the development of computed tomography (CT) technology, coronary CT angiography can be acquired with low doses of radiation and contrast agent without a loss of diagnostic performance. The primary objective of the CONCENTRATE study is to prove the noninferiority of the enhancement effect of low-concentration contrast agents compared to a high-concentration contrast agent of the coronary artery and myocardium with coronary CT angiography.

**Methods/Design:**

The CONCENTRATE study is a prospective, multicenter, noninferiority, randomized trial evaluating the enhancement effect of low-concentration contrast agents (270 and 320 mg iodine/ml) compared with a high-concentration contrast agent (370 mg iodine/ml) in the coronary artery and myocardium of coronary artery CT angiography. The primary efficacy measurement is the enhancement of coronary arteries as measured in Hounsfield units. The target population comprises 318 patients with suspected coronary artery disease who have been referred for clinically indicated nonemergent coronary CT angiography. Eligible participants are randomized for three different concentrations of the contrast agent in a 1:1:1 allocation ratio to one of three arms. The CONCENTRATE trial is a double-blind study, where the subjects and the outcome assessor are blinded to the concentration of the contrast agent used for coronary the CT angiography. Eight clinical sites in Korea are participating in this trial.

**Discussion:**

The CONCENTRATE study will determine whether low-concentration contrast agents are able to provide diagnostic image quality in coronary CT angiography.

**Trial registration:**

NCT02549794. Registered on 14 September 2015.

**Electronic supplementary material:**

The online version of this article (doi:10.1186/s13063-016-1441-y) contains supplementary material, which is available to authorized users.

## Background

Due to developments in computed tomography (CT) technology, cardiac CT has become very useful as a noninvasive examination technique in the diagnosis of obstructive coronary artery disease (CAD), and the accuracy has increased to more than 90 % [1–5]. Specifically, cardiac CT plays a gatekeeper role in reducing invasive cardiac angiography implemented solely for the purpose of diagnosis [6]. However, cardiac CT also has disadvantages, particularly the exposure of patients to radiation and iodine contrast agent. Consequently, considerable effort has been devoted to identifying ways to reduce the radiation exposure and the amount of contrast agent used. A recently introduced method uses a combination of a scan with a low tube-based potential and iterative image reconstruction to reduce both the radiation dose and the amount of contrast agent used for coronary CT angiography [7]. According to recent studies of values from this combined method, the signal-to-noise ratio (SNR) and contrast-to-noise ratio (CNR), which represent image quality, are higher compared to the standard method [8, 9]. The standard method utilizes a scan with higher tube potential according to patient body mass index (BMI) and image reconstruction by makeshift filtered-back projection under conditions using the same amount of contrast agent. Therefore, the amount of contrast agent can be reduced while achieving the same contrast effect due to the advantage of the increased effect of contrast enhancement provided by the low tube potential. Therefore, efficacy studies using low-concentration contrast agents along with low tube potential are being performed [10].

The CONCENTRATE study intends to prove that, compared to the combined method using the makeshift filtered-back projection image reconstruction and standard image acquisition according to BMI and with a standard high-concentration contrast agent, the image quality does not deteriorate as a result of the combination of a scan with low tube potential and the iterative image reconstruction method with low-concentration contrast agents.

### Study objectives

#### Primary objective

The primary objective of the CONCENTRATE study is to determine the noninferiority of the contrast enhancement of cardiac CT with low-concentration contrast agents compared to that with high-concentration contrast agent.

#### Secondary objective

The secondary objective of the CONCENTRATE study is to determine the diagnostic accuracy of coronary CT angiography in the identification of anatomically obstructive CAD with low-concentration contrast agent compared with invasive coronary angiography (ICA) as the reference standard and to compare it to the accuracy achieved with a high-concentration contrast agent.

### Primary hypothesis

We hypothesized that the use of low-concentration contrast agent for coronary CT angiography and myocardial perfusion would not be inferior to the use of a high-concentration contrast agent in the enhancement effect in the coronary artery and in the myocardium.

## Methods/Design

### Trial design

**Fig. 1:**
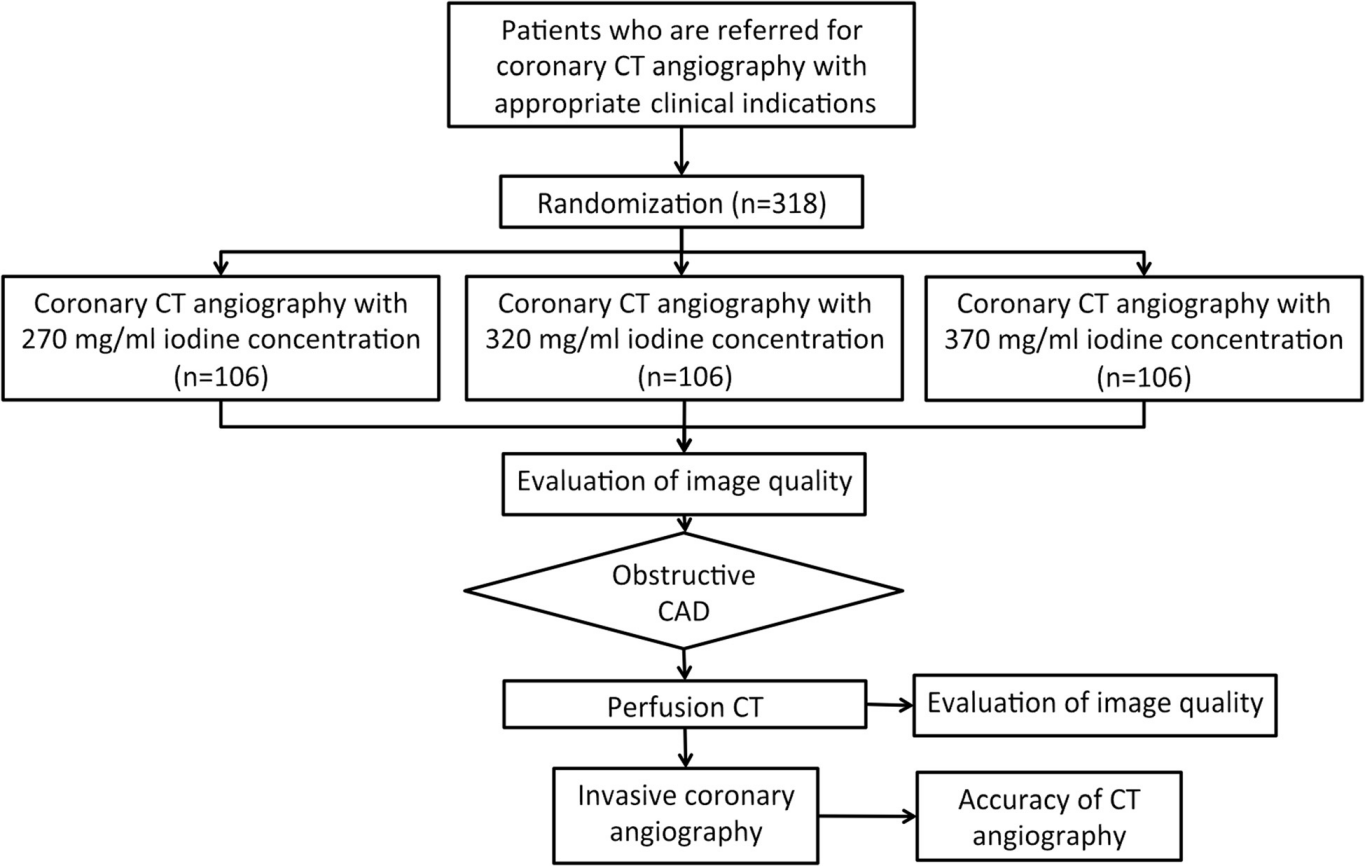
CONCENTRATE study workflow. CT, computed tomography; CAD, coronary artery disease

The CONCENTRATE study is a prospective, multicenter, noninferiority, randomized trial evaluating the enhancement effect of low-concentration contrast agents consisting of 270 and 320 mg iodine/ml (mgI/ml), compared with a high-concentration contrast agent that contains 370 mgI/ml in the coronary artery and myocardium in coronary artery CT angiography. The target population includes patients with suspected CAD who have been referred for clinically indicated nonemergent ICA. Eligible participants are randomized for three different contrast agent concentrations in a 1:1:1 allocation ratio to one of three arms of the trial. The CONCENTRATE trial is a double-blind study, where the subjects and the outcome assessor are blinded to the concentration of the contrast agent used for coronary CT angiography. Eight clinical sites in Korea are participating in this trial. Every clinical site requires the approval of the site’s Institutional Review Board. The study protocol and the informed consent form should be approved by the Institutional Review Board at each participating site. A list of the Institutional Review Boards and the status of their respective approvals are provided in an additional file (see Additional file 1). Standard protocol items and organizational structures are provided as additional files (see Additional files 2 and 3, respectively). A flow chart of the study is provided in Fig. 1. The informed consent will be obtained from all participants (see Additional file 4).

### Participants

Suitable participants include patients ≥ 20 years of age who have requested coronary CT angiography to assess clinical disease. The exclusion criteria include the following: 1) subjects suspected of having myocardial infarction, unstable angina pectoris, or coronary artery disease; 2) subjects with heart attack within 40 days prior to the CT scan; 3) subjects with a diagnosed complicated heart anomaly; 4) BMI > 35 kg/m^2^; 5) serum creatinine ≥ 1.5 mg/dl of renal insufficiency; 6) pregnant subjects; 6) subjects with a history of hypersensitivity reactions to contrast agents; 7) subjects with contraindications to the use of nitroglycerine; 8) subjects who plan to participate or enroll in other randomized clinical trials for cardiovascular disease; or 9) subjects with contraindications to the use of adenosine (e.g., bronchial asthma, 2–3 degree atrioventricular block, sick sinus syndrome, systolic blood pressure (SBP) less than 90 mmHg, recent prescribed history of dipyridamole, or hypersensitivity to adenosine) (Table 1). Patients who meet the selection criteria are registered by acquiring informed consent at the time an examination is ordered and during outpatient treatment by the investigators. Time schedule, interventions, assessments, and visits for participants are provided in a table (Table 2).

**Table 1:** Inclusion and exclusion criteria

Inclusion criteria • Adults at least 20 years old • Subject who requested a coronary CT angiography to assess clinical disease Exclusion criteria • Subjects suspected of having myocardial infarction, unstable angina pectoris, or coronary artery disease • Subjects who experienced heart attack within 40 days prior to the CT scan • Subjects with a diagnosed complicated heart anomaly • BMI > 35 kg/m^2^ • Serum creatinine ≥ 1.5 mg/dl • Pregnant subjects • Subjects with a history of hypersensitivity reaction to contrast agents • Subjects with contraindications to the use of nitroglycerine • Subjects who plan to participate or enroll in other randomized clinical trials for cardiovascular disease. • Subjects with contraindications to the use of adenosine (e.g., bronchial asthma, 2–3 degree AV block, sick sinus syndrome, SBP less than 90 mmHg, recent prescribed history of dipyridamole, or hypersensitivity to adenosine)	

*CT* computed tomography, *BMI* body mass index, *SBP* systolic blood pressure

**Table 2:** Schedule of forms and procedures

	Baseline	CT angiography	CT perfusion	Coronary angiography	Study evaluation
	Visit 1	Visit 2	Visit 3	Visit 4	
Screening	x				
Demographic	x				
Medical history	x				
Randomization	x				
Vital signs		x			
Laboratory test	x				
Vital signs 2		x			
CT angiography		x			
Vital signs 3			x		
CT perfusion			x		
Coronary angiography				x	
Study evaluation					x

### Randomization

All enrolled subjects are randomly assigned to one of three concentrations of contrast agent in a 1:1:1 ratio based on each trial site. We use concealed allocation and an adequate computer-generated allocation sequence to avoid selection bias. Thus, neither the patient nor the outcome assessor knows to which group the patient is allocated. Therefore, if unblinding is deemed necessary, any of the investigators, coordinators, or CT operators can provide the information of the contrast agent used. If the assessors of the outcome learn this information, they should report this on the corresponding case report form.

### Interventions

Three different concentrations of contrast agent are randomly assigned to patients undergoing CT coronary angiography. A high tube potential is used for CT scans with the high-concentration contrast agent (370 mgI/ml), whereas a tube potential that is 20 kVp lower is used with the low-concentration contrast agents (270 mgI/ml and 320 mgI/ml).

#### Preparation of patients

If participants do not have contraindications for the use of the nitroglycerine, they receive sublingual nitroglycerine before coronary CT angiography. If a participant’s heart rate is equal to or greater than 60 beats per min, a beta-blocker is administered. If > 50 % stenosis is apparent on the coronary CT angiography, participants will undergo stress perfusion CT. In these cases, nitroglycerine and beta-blockers are not used. Separate intravenous lines are secured for the injection of adenosine and contrast media.

#### Contrast agents

Three contrast agents with different iodine concentrations (Visipaque 270, iodixanol 270 mgI/ml; GE Healthcare, Giles St Chalfont, United Kingdom vs. Visipaque 320, iodixanol 320 mgI/ml; GE Healthcare, Giles St Chalfont, United Kingdom vs. Pamiray 370, iopamidol 370 mgI/ml; Dongkook Pharma, Seoul, Korea) will be compared in this study. The participants are randomly assigned to different contrast agents in the same proportions. All contrast agents will be maintained by the same storage process, based on hospital systems, as used in general CT examinations. Each contrast agent will be administered via the antecubital vein of patients in the same triphasic injection. In the first phase, a 50-ml bolus of contrast agent will be injected at 5 ml/s. Then, 50 ml of mixed saline with 30 ml iodine and 20 ml saline will be injected into patients at 5 ml/s, followed by 40 ml of saline chaser. The total injected volume of the contrast agent is 80 ml.

#### Coronary CT protocol

All coronary CTA studies are acquired with a multidetector CT scanner (Discovery HD 750; Gemstone Spectral Imaging, GE Healthcare, Milwaukee, WI, USA). During scanning, participants hold their breath and are still. To obtain better image quality for each contrast agent, scans are performed with different protocols depending on whether high- or low- concentration contrast agents are used. If a high-concentration contrast agent (iopamidol 370 mgI/ml) is used, scans are conducted at 120 kVp for higher BMI (27 < BMI < 35) patients and at 100 kVp for lower BMI (15 < BMI ≤ 27) patients, with adjusted mAs (BMI-based tube potential selection). If a low-concentration contrast agent (iodixanol 270 mgI/ml or iodixanol 320 mgI/ml) is used, scans are conducted 20 kVp lower than in the BMI-based tube potential protocol: that is, at 100 kVp for higher BMI and 80 kVp for lower BMI. A beta-blocker is used for patients with a heart rate higher than 60 beats per minute. Scans initiate a bolus tracking method with a 4.8-s delay after reaching 100 Hounsfield units (HU) in the ascending aorta enhancement.

#### Myocardial perfusion CT

Stress myocardial perfusion CT will be performed in patients with at least one segment of greater than 50 % stenosis on coronary CT angiography. A static CT perfusion protocol will be used, and stress will be induced by infusing 140 mcg/kg/min adenosine under ECG monitoring for up to 6 min. The randomly assigned contrast agent, which was used for coronary CT angiography in the patient, will be injected 4 min 30 s after the adenosine injection, using the same injection method for contrast agent and image acquisition settings as used for the coronary CT angiography with the only difference being an additional scan delay of 2 s.

#### Image reconstruction

All images will be reconstructed using an iterative reconstruction algorithm at 50 % adaptive statistical iterative reconstruction (ASIR, GE Healthcare, Waukesha, Wis, USA).

#### Evaluation of radiation dose

The dose-length product (DLP) from each shooting will be collected. The effective radiation dose (mSv) will be calculated using a conversion factor of 0.014 mSv mGy^-1^ cm^-1^.

#### Retention

This study does not follow the participants’ outcome. Therefore, no specific plan has been developed to promote participant retention.

### Efficacy analysis

#### Primary efficacy analysis

The primary efficacy measurement is the HU value of the coronary arterial lumen acquired from the comparison of the image quality from the three different protocols of contrast media in coronary CT angiography. The coronary artery is divided into 17 segments according to the modified American Heart Association (AHA) classification, and luminal enhancement is measured for each segment with avoidance of the borderline between the lumen and the vessel wall or epicardial tissue, artifacts, or calcification. Measurement is performed on three different points in each segment and the average value is used for the HU value of each segment. The mean HU value of left main artery and proximal right coronary artery will be compared between the different arms of the trial.

#### Secondary efficacy analysis

Qualitative evaluation of the image quality of the coronary artery will be performed. Two experienced observers will review all coronary CT images and score the image quality for each segment with a 4-point grading system on visual assessment: Grade 1, nondiagnostic; Grade 2, reduced image quality; Grade 3, nonlimiting artifacts; and Grade 4, complete absence of motion artifacts with good attenuation of the vessel lumen and clear delineation of the vessel walls with the additional ability to assess luminal stenosis.

Quantitative evaluation of image quality of perfusion CT will be performed. The myocardium will be divided into 16 segments according to AHA classification, and the HU value of each segment will be measured. Mean HU value, SNR, and CNR of myocardial enhancement will be compared between the different arms of the trial.

Diagnostic accuracy of coronary CT angiography compared to invasive coronary angiography will be calculated. Segment-basis analysis, sensitivity, specificity, positive predictive value, negative predictive value, and accuracy of CT angiography for diagnosis of the presence of CAD defined by more than 50 % diameter stenosis compared to invasive coronary angiography will be calculated and compared between the different concentrations of contrast agent by means of a generalized estimating equation based on a binary logistic model.

Quantitative evaluation of image quality of coronary artery will be performed on a per-vessel and per-segment basis.

### Statistical methods

#### Sample size and power calculation

The noninferiority margin was justified by an indirect confidence interval approach using the point estimate because the constancy assumption was not applicable in this trial. In a previous study [11], the reduction rate between iopamidol 370 mgI/ml and iodixanol 320 mgI/ml was 18 % (from 439.96 to 362.06 HU), and we assumed that the HU value between the low-concentration and high-concentration groups would be the same. Consequently, we estimate 9 % (0.5 × (18 % - 0 %)) of the reported mean HU [10] to be the noninferiority margin, allowing a loss of less than 50 % of the active control effect, which corresponds to a noninferiority margin of 51.26 HU [12]. Clinically, 250–300 HU is considered sufficient enhancement for coronary angiography [13]. Therefore, a reduction of 51.26 HU from 439.96 HU, which results in 388.7 HU, is clinically acceptable. For the mean contrast enhancement as an attenuation value of CT (HU) in the noninferiority test, we assumed same mean HU among the three groups and a standard deviation of 118.93 for the HU based on a previous study [10]. With these assumptions and a 10 % dropout rate, 106 subjects per group are needed to obtain 80 % statistical power with a corrected two-sided α ≒ 0.0167 (=0.05/3).

#### Primary statistical analysis

Per-protocol analysis will be performed primarily; an additional intention-to-treat analysis will be also performed. The 98.33 % confidence interval (CI) for the difference of mean contrast enhancement in the ROIs will be calculated. The noninferiority of the low-dose group compared to the high-dose group will be demonstrated if the lower bound of the two-sided 98.33 % CI lies above the pre-specified noninferiority margin. Because missing values are expected for 5 % or fewer of the participants, we have planned complete-case analysis for the primary analysis. To account for multiple observations per patient in secondary analysis, we will use a linear mixed model, including fixed effects for the group and random intercepts for the patient. Patients with a missing observation in some vessel or segment will be included in the per-vessel and per-segment analysis by using the linear mixed model. Analysis of covariance (ANCOVA) with BMI as a covariate will be used to compare the mean HU of the myocardium on static perfusion CT. Inter-reader agreement for the assessment of image quality will be evaluated using a linearly weighted kappa statistic. Analysis of variance (ANOVA) will be used to compare mean changes in heart rate before and after CT examinations among the three groups. To compare the diagnostic accuracy of invasive coronary angiography for diagnosis of significant coronary artery stenosis with more than 50 % stenosis among the three groups, logistic regression analysis using a generalized estimating equation (GEE) will be used to account for the correlation among multiple segments within the same subject. All statistical analyses will be performed using SAS (SAS Ver. 9.2; SAS Institute, Cary, NC, USA) and two-sided *P* values less than 0.05 will be considered statistically significant.

### Data management

The data-coordinating center in Severance Hospital is collecting data through a secure Internet connection to the central server and monitoring the overall dataset. At the start of the trial, the monitors conducted a tutorial on the web-based data entry system and the image upload system. They will audit the overall quality and integrity of the data regularly every 6 months and, if necessary, contact the site investigator and coordinator to review and confirm the correctness of the data with source data in compliance with the protocol. The monitors will verify that all adverse events were documented in the correct format and are consistent with protocol definition. The monitor conducts the monitoring procedure independently from the investigators and the sponsor. The primary and secondary endpoints in this trial include only the image quality and not the patients’ clinical outcomes. If the quality is not enough to determine significant coronary artery disease due to insufficient enhancement from a low-concentration contrast agent, repeat examination with a high-concentration contrast agent can be used at the site investigator’s discretion. Therefore, the conduct of an interim analysis is not needed to evaluate any potentially important reasons to modify or discontinue the trial. Adverse effects and serious adverse events will be recorded in the case report form. A summary of adverse effects and serious adverse effects will be immediately forwarded the independent Institutional Review Board and local health authorities, according to local regulations.

## Discussion

The CONCENTRATE study is a prospective, multicenter, noninferiority, randomized trial evaluating the enhancement effect of two low-concentration contrast agents compared with the high-concentration contrast agent recommended for sufficient opacification in coronary CT angiography, according to established guidelines [14].

The accuracy of coronary CT angiography in the diagnosis of coronary artery stenosis is affected by image quality, which is dependent on the CNR. To achieve a high CNR, high-concentration contrast agents are usually recommended and have been widely used in everyday clinical practice as a standard protocol [14]. According to a study that compared two contrast agents with different concentrations (400 mgI/ml vs. 320 mgI/ml), coronary arterial enhancement was higher when the high-concentration contrast agent was used and higher enhancement levels were found to be associated with lower numbers of inadequately visualized segments [11]. However, in another study that compared four different concentrations (370, 350, 320, and 270 mgI/ml), image quality grade was higher with low-concentration contrast agents, although high-concentration contrast agents showed greater vascular enhancement [15]. The fact that heart rate variability was lower with low-concentration contrast agents, which were all iso-osmolar agents, whereas the high-concentration contrast agents were all low-osmolar agents, might partially account for the higher image quality obtained with lower-concentration-contrast agents in this study. The lowest enhancement of 369.1 ± 85.4 HU obtained with the lowest concentration of 270 mgI/ml in this study could be considered adequate because adequate intra-arterial enhancement for coronary CT angiography has been considered to require more than 250 HU according to previous reports [13, 14].

In this regard, an effort to reduce the amount of iodine should be considered to reduce the probability of contrast-induced nephropathy in patients at risk, as long as diagnostic image quality is maintained [16, 17]. When using low-concentration contrast agents, a combination of low tube potential and iterative reconstruction would be helpful for maintaining high vascular enhancement and image quality. A lower tube potential has the advantages of reducing radiation dose and improving image contrast. However, using a lower tube potential reduces X-ray penetration and increases image noise. Iterative reconstruction is a solution for this problem because it improves image quality by reducing image noise. The combination of a lower tube potential and iterative image reconstruction does not result in a deterioration of image quality and diagnostic accuracy [8, 9]. A single center study tested the feasibility of this combination with a low-concentration (270 mgI/ml) contrast agent compared to a high-concentration contrast agent (370 mg iodine/ml) in coronary CT angiography and demonstrated that a low-concentration contrast agent maintained the contrast enhancement without impairing image quality [10]. The CONCENTRATE trial has been designed to validate this finding as a multicenter study to limit possible bias and secure maximum generalizability.

The CONCENTRATE study will determine whether low-concentration contrast agents are able to provide diagnostic image quality on coronary CT angiography. Because the CONCENTRATE trial will only measure the coronary artery lumen and the myocardium in coronary CT angiography, the results of the CONCENTRATE trial should be further validated for other applications of cardiac CT, including in the evaluation of plaque, in-stent restenosis, and image-based fractional flow reserve. With expanding options for technology, such as feasibly scanning with 70 kVp, and more sophisticated iterative reconstruction methods, CT protocols should be further optimized to apply the results of the CONCENTRATE trial in the real world.

## Trial status

Recruitment commenced in July 2015.

## Abbreviations

AHA, American Heart Association; BMI, body mass index; CAD, coronary artery disease; CNR, contrast-to-noise ratio; CT, computed tomography; ECG, electrocardiography; HU, Hounsfield Unit; ICA, invasive coronary angiography; SNR, signal-to-noise ratio

## Additional files

Additional file 1: List of Ethics Committees and Status of Approval. List of the ethics committees of all eight participating centers and the status of approval of the study at the time of submission. (DOCX 13 kb) Additional file 2: SPIRIT checklist 2013. (DOCX 52 kb) Additional file 3: Organizational structure. (DOCX 86 kb) Additional file 4: Model consent form. (DOCX 160 kb)
