# Supplementary material for: Comparison of coronary computed tomography angiography image quality with high- and low-concentration contrast agents (CONCENTRATE): study protocol for a randomized controlled trial
Source: Trials. 2016 Jul 15;17:315. doi: 10.1186/s13063-016-1441-y (PMC4946231; doi:10.1186/s13063-016-1441-y)
Supplement: Additional file 1: — List of Ethics Committees and Status of Approval. List of the ethics committees of all eight participating centers and the status of approval of the study at the time of submission. (DOCX 13 kb) [file 13063_2016_1441_MOESM1_ESM.docx]

**List of Ethics Committees and Status of Approval**

As of Oct 26, 2015, the status of approval in each investigating site as follows.

- Institutional Review Board, Human Research Protection Center, Severance Hospital, Yonsei University Health System; approved (4-2015-0173)
- Institutional Review Board, Clinical Trial Center, Chonnam National University Hospital; approved (CNUH-2015-224)
- Institutional Review Board, Clinical Trial Center, Pusal National University Hospital; approved (04-2015-023)
- Institutional Review Board, Asan Medical Center; approved (2015-0942)
- Institutional Review Board, Office of Human Research Protection Program, Seoul St. Mary’s Hospital, Catholic Medical Center; approved (XC15OIMI0031K)
- Institutional Review Board, Office of Human Research Protection Program, Uijeongbu St. Mary’s Hospital, Catholic Medical Center; approved (XC15OIMI0031U)
- Institutional Review Board, Office of Human Research Protection Program, Yeouido St. Mary’s Hospital, Catholic Medical Center; approved (XC15OIMI0031S)
- Institutional Review Board, Office of Human Research Protection Program, Bucheon St. Mary’s Hospital, Catholic Medical Center; approved (XC15OIMI0031H)
