# Supplementary material for: Comparison of coronary computed tomography angiography image quality with high- and low-concentration contrast agents (CONCENTRATE): study protocol for a randomized controlled trial
Source: Trials. 2016 Jul 15;17:315. doi: 10.1186/s13063-016-1441-y (PMC4946231; doi:10.1186/s13063-016-1441-y)
Supplement: Additional file 3: — Organizational structure. (DOCX 86 kb) [file 13063_2016_1441_MOESM3_ESM.docx]

Organisational structures and responsibilities of the CONCENTRATE trial

**Principal Investigator, Research Physician, and Administrator**Design and conduct of the CONCENTRATE trial.
Study planning
Preparation of protocol and revisions
Preparation of investigators brochure (IB) and CRFs [Case Report Forms]
Organising steering committee meetings
Publication of study reports
Organisation of steering committee meetings
Responsible for trial master file
Budget administration and contractual issues with individual centers
Randomisation

**Steering committee (SC)**Agreement of final protocol
All sites’ principle investigators will be steering committee members.
Recruitment of patients and liaising with principle investigator
Reviewing progress of study and if necessary agreeing changes to the protocol and/or investigators brochure to facilitate the smooth running of the study.

**Data Coordinating Center**Maintenance of trial IT system and data entry
Data verification

**Data Safety Monitoring Board**
Audit the overall quality and integrity of the data regularly every 6-months
Verify that all adverse events were documented in the correct format, and are consistent with protocol definition.
Conducts monitoring procedure independently from the investigators and the sponsor

**Sites’ Investigators**In each participating a principle investigator will be identified, to be responsible for identification, recruitment, data collection and completion of CRFs, along with follow up of study patients and adherence to study protocol.
