# Supplementary material for: Comparison of coronary computed tomography angiography image quality with high- and low-concentration contrast agents (CONCENTRATE): study protocol for a randomized controlled trial
Source: Trials. 2016 Jul 15;17:315. doi: 10.1186/s13063-016-1441-y (PMC4946231; doi:10.1186/s13063-016-1441-y)
Supplement: Additional file 4: — Model consent form. (DOCX 160 kb) [file 13063_2016_1441_MOESM4_ESM.docx]

**AN AGREEMENT TO BE IN A RESEARCH STUDY**

**INFORMED CONSENT DOCUMENT**

**Sponsor:** Severance Hospital

**City and State:** Seoul, South Korea

**Protocol Number and Title:** Comparison of coronary computed tomography angiography image quality with high- and low-concentration contrast agents: study protocol for a randomized controlled trial (CONCENTRATE)

**Study Doctor:** «Investigator»

**Address of Study Site(s):**  «Study_Site_1»

«Address_1»

«City _Zip_1»

**24-Hour Telephone Number:** «M_24_Hour_Number_1»

**INTRODUCTION**

You are invited to consider participating in a research study.

You are being asked to participate in this study because your physician believes that you require coronary computed tomography angiography. Advanced non-invasive cardiac technologies now exist that can examine the coronary arteries and heart muscle to determine whether you have blockages in the heart arteries or decreased blood flow to the heart muscle, respectively. This test is called a coronary computed tomographic angiogram (CCTA). CCTA can identify obstructive coronary artery disease with high accuracy. CCTA requires administration of contrast agent. In this research study, we will test whether low-concentration contrast agents are able to provide diagnostic image quality in CCTA.

Please take your time to make your decision. It is important that you read and understand several general principles that apply to all who take part in our studies:

(a) Taking part in the study is entirely voluntary.

(b) Personal benefit to you may or may not result from taking part in the study, but knowledge gained from your participation may benefit others.

(c) You may decide not to participate in the study or you may decide to stop participating in the study at any time without loss of any benefits to which you are entitled.

(d) You must be honest and complete in providing your medical history. Giving false, incomplete, or misleading information about your medical history, including past and present drug use, could have very serious health consequences.

The purpose and nature of the study, possible benefits, risks, and discomforts, other options, your rights as a participant, and other information about the study are discussed below. Any new information discovered which might affect your decision to participate or remain in the study will be provided to you. You are urged to ask any questions you have about this study with members of the research team. You should take whatever time you need to discuss the study with your personal physician and family. The decision to participate or not to participate is yours. When we have answered all of your questions, you can decide if you want to be in the study or not. This process is called "informed consent." We will give you a signed and dated copy of this form for your records. The consent form may contain words that you do not understand. Please ask the study doctor (researcher) or the study staff to explain any words or information that you do not clearly understand. You may take home an unsigned copy of this consent form to think about or discuss with family or friends before making your decision.

This research study is funded by GE Healthcare. They provide funding to cover part of the costs of conducting this study.

If you decide to participate, please sign and date where indicated at the end of this form.

**WHY IS THE STUDY BEING DONE?**

The purpose of this study is to demonstrate we will test whether low-concentration contrast agents are able to provide diagnostic image quality in CCTA. If it is true, we may use reduced dose of contrast agent for CCTA.

**HOW MANY PEOPLE WILL TAKE PART IN THE STUDY?**

About 318 participants, 20 years and older, will take part in this study.

**WHAT IS INVOLVED IN THE STUDY?**

For this study, you will undergo a CCTA. A CCTA is similar to an x-ray and involves the use of iodine contrast dye. This is a non-invasive procedure, and the CCTA scanner used in this study is Korea FDA approved. A heart rate lowering medication called Beta Blocker is normally used during a CCTA.

The research conducted during this study will be the collection of information from you and your medical records at the time of your procedure. There is no follow up for this research study.

Please tell the study doctor or study staff of any medications you are taking. In addition, if you are taking any over-the-counter drugs or herbal supplements which you have obtained from the drug store, grocery store, etc., you should tell the study doctor or study staff.

**HOW LONG WILL YOU BE IN THE STUDY?**

You will be in the study until the performance of the CCTA.

We will record information from your medical record about the diagnosis and treatment of your heart condition. This health information and all health information that we collect during the study will be kept as study information.

You can stop participating at any time. However, if you decide to stop participating in the study, we encourage you to talk to the study doctor and your regular doctor first.

There are no consequences of sudden withdrawal from the study.

**WITHDRAWAL BY STUDY DOCTOR, PHYSICIAN, IRB**

The study doctor, physicians, or IRB may stop the study or take you out of the study at any time should they judge that it is in your best interest to do so, if you experience a study-related injury, if you need additional or different medication, or if you do not comply with the study plan. They may remove you from the study for various other administrative and medical reasons. They can do this without your consent.

If you withdraw from the study, no new data about you will be collected for study purposes. All data that have already been collected for study purposes will be shared with the study sponsor.

**WHAT ARE THE RISKS OF THE STUDY?**

You are undergoing this imaging procedure because your physician has judged that it is medically necessary. The risks (including the contrast dye used) listed below are not increased by the study procedures.

Your decision to be in the study has two main impacts:

- The standard imaging procedure usually calls for administering high-concentration contrast agent to possibly maintain the best image quality. In the study, you will be randomly assigned to either high- or low-concentration contrast agent to use. When using low-concentration contrast agent, the image quality may be affected. To improve image quality to compensate for this, we will use two methods; lower tube potential by 20 kVp and iterative reconstruction of images. According to single center studies, use of low-concentration contrast agent is not inferior regarding image quality.

Also, some of the questions that are asked to you may be embarrassing to you. You may refuse to answer any of the questions.

Procedure Risks: These risks will be discussed with you by the study doctor and/or your regular doctor.

Risks and side effects related to the procedures we are studying include:

- Possible side effects – warm feeling or flushed feeling during contrast injection
- Rare side effects – contrast allergy; contrast induced nephropathy (deterioration of kidney function due to contrast exposure); hives (for those allergic to contrast dye)
- Very rare side effects: major bleeding, damage to an artery, blood clot, heart attack, stroke, irregular heart beat (may be life-threatening)
- There may also be side effects, other than listed above that we cannot predict, some of which may be life-threatening. Other drugs will be given to make side effects that occur less serious and less uncomfortable. In some cases side effects can be serious, long lasting or permanent.

For more information about risks and side effects, ask the study doctor or study staff at the phone number listed on the first page of this consent document.

You must tell the study doctor or study staff about all side effects that you have. If you are not honest about your side effects, it may not be safe for you to stay in the study.

**ARE THERE ANY BENEFITS TO TAKING PART IN THE STUDY?**

Since this study does not provide treatment, there is no direct benefit to you. Information learned from the study may help other people in the future.

**WHAT OTHER OPTIONS ARE THERE?**

Taking part in this study is your choice. You may choose either to take part or not to take part in the study. You have the right to leave this study at any time. If you do not want to be in the study, there will be no penalty to you, and you will not lose any benefits to which you are otherwise entitled.

If you wish to leave this study, please call the Study Doctor or study staff at the telephone number listed on the first page of this consent document to schedule study exit procedures.

**WHAT ABOUT CONFIDENTIALITY?**

We are required by national regulations to put information about your participation into a medical database. The information in this medical database will include:

• Name of the study

• The name of the researcher

• The name of the study coordinator

• Contact phone number for the study

• Contact email address for the study

• Emergency phone number for the study

• Expected start and end dates for your time in the study

• Whether there are healthy volunteers in the research

Information about your research procedures and test results may also be put in your medical record. This will include:

• Imaging test results

• Your medical history, including medications and symptoms that you may feel

Efforts will be made to protect your medical records and other personal information to the extent allowed by law. However, we cannot guarantee absolute confidentiality.

Medical records of research study participants are stored and kept according to legal requirements. You will not be identified personally in any reports or publications resulting from this study. The study information about you will be numbered and linked to your name. We will keep a master list indefinitely.

We will put a copy of this consent form in your medical record. We will also put copies of test and exam results from this study into your medical record if the results are important for your medical care.

Organizations that may request to inspect and/or copy your research and medical records for quality assurance and information analysis include groups such as:

The study doctor, the study sponsor, Korean Food and Drug Administration (KFDA), the medical center, the Institutional Review Board (IRB), and all national research oversight agencies may have access to the study records.

If information about your participation in this study is stored in a computer, we will take the following precautions to protect it from unauthorized disclosure, tampering, or damage:

We will keep a database on a computer that has anonymized all individuals enrolled into the study. The database will be accessible only by password. Only the researchers of the study will be allowed to see the information.

A description of this clinical trial will be available on http://www.ClinicalTrials.gov, as required by U.S. Law. This Web site will not include information that can identify you. At most, the Web site will include a summary of the results. You can search this Web site at any time.

**WHAT ARE THE COSTS?**

The cost of the CCTA will be covered by the study sponsor. Associated medical costs will be billed to you or your insurance company. You or your insurance company will be billed for continuing medical care and/or hospitalization that are not a part of the study.

The study doctor or study staff can provide you with more detailed information.

**RESEARCH RELATED INJURY**

The sponsor will not offer to pay for care necessitated by a research related injury.

In accordance with national regulations, we are obligated to inform you about the Medical Center’s policy in the event injury occurs. If, as a result of your participation, you experience injury from known or unknown risks of the research procedures as described, immediate medical care and treatment, including hospitalization, if necessary, will be available and the costs will be billed to the study participant. No other form of compensation is offered. Please be aware that some insurance plans may not pay for research-related injuries. You should contact your insurance company for more information. You should not expect anyone to pay you for pain, worry, lost income, or non-medical care costs that occur from taking part in this research study.

**PAYMENT FOR PARTICIPATION**

**WHAT ARE YOUR RIGHTS AS A PARTICIPANT?**

Taking part in this study is voluntary. You may choose to not take part in the study or to leave the study at any time. If you choose to not participate in the study or to leave the

study, your regular care will not be affected nor will your relations with the Medical Center, your physicians, or other personnel. In addition, you will not lose any of the benefits to which you are entitled.

We will tell you about new information that may affect your health, welfare, or participation in this study.

**LEGAL RIGHTS**

You do not lose any legal rights by signing this consent document. The above statement, “What Are Your Rights as a Participant” does not stop you from getting legal help in case of negligence.

**WHO DO YOU CALL IF YOU HAVE QUESTIONS OR PROBLEMS?**

You may contact the Study Doctor or study staff at the phone number listed on the first page of this consent document:

- - for answers to questions, concerns, or complaints about this research study
  - to report a research related injury, or
  - for information about study procedures.

If you need medical attention please go to the nearest emergency room.

You may contact Severance Hospital if you:

- would like to speak with someone not related to the research,
- have questions, concerns, or complaints regarding the research study, or
- have questions about your rights and welfare as a research participant.

Chairman, Severance Institutional Review Board

50-1 Yonsei-ro, Seodaemun-gu

Seoul, South Korea 03722

Or you can call: 02-2228-0430 between 8:00 AM and 5:00 PM

If you would like additional information, you may visit Severance’s website at [eirb.yuhs.ac.](http://www.rcrcirb.com)

Severance Hospital’s Institutional Review Board has approved this study and this informed consent document. Institutional Review Board is a group of scientific and non-scientific people who review, and approve or disapprove research involving people by following the national regulations. This group is also required by the national regulations to do periodic review of ongoing research studies.

**SUBJECT’S STATEMENT**

This consent document contains important information to help you decide if you want to be in this study. If you have any questions that are not answered in this consent document, please ask the person explaining this document or one of the study staff.

By consenting to participate you agree that you have been given a copy of all pages of this consent document. You have had an opportunity to ask questions and received satisfactory answers to all your questions about this study. You understand that you are free to leave the study at any time without having to give a reason and without affecting your medical care. You understand that your study-related medical records may be reviewed by government authorities.

If you do not agree with the statement above, you should not sign this informed consent document.

____________________________________________________

Printed Name of Adult Participant

____________________________________________________ ____________ Signature of Adult Participant Date

**DO NOT SIGN AFTER ********************

**RESEARCHER’S STATEMENT**

I have fully explained this study to the subject. As a representative of this study, I have explained the purpose, the procedures, the benefits and risks that are involved in this research study. Any questions that have been raised have been answered to the individual’s satisfaction.

____________________________________________________

Printed Name of Person Explaining Informed Consent Document

(Principal Investigator or Co-investigator)

____________________________________________________ ____________ Signature of Person Explaining Informed Consent Document Date

You will be given a signed and dated copy of this informed consent document to keep.
